# Intravenous Lidocaine for Postoperative Pain and Recovery After Robotic Prostate Adenomectomy: A Retrospective Observational Cohort Study

**DOI:** 10.3390/medicina61112045

**Published:** 2025-11-16

**Authors:** Georgiana Maria Popa, Simona-Alina Abu-Awwad, Ahmed Abu-Awwad, Carmen-Ioana Marta, Erika Bimbo-Szuhai, Mihaela Gabriela Bontea, Adrian Gheorghe Osiceanu, Anca Mihaela Bina, Cristian Mihai Moisa Cezar, Ciprian Dumitru Puscas, Mihai O. Botea

**Affiliations:** 1 Pelican Hospital, Corneliu Coposu Street 2, 410450 Oradea, Romania; pascalau.georgianamaria@student.uoradea.ro (G.M.P.); bimboszuhai.erika@didactic.uoradea.ro (E.B.-S.); mbotea@uoradea.ro (M.O.B.); 2 Department of Surgical Disciplines, Faculty of Medicine and Pharmacy, University of Oradea, 1st December Square 10, 410073 Oradea, Romania; 3 Doctoral School of Biomedical Sciences, University of Oradea, 410087 Oradea, Romania; moisa.cezarcristianmihai@student.uoradea.ro; 4 Department of Obstetrics and Gynecology, “Victor Babes” University of Medicine and Pharmacy Timisoara, Eftimie Murgu Square 2, 300041 Timisoara, Romania; alina.abuawwad@umft.ro (S.-A.A.-A.); carmen.marta@umft.ro (C.-I.M.); lungu.anca@umft.ro (A.M.B.); 5 Clinic of Obstetrics and Gynecology, “Pius Brinzeu” County Clinical Emergency Hospital, 300723 Timisoara, Romania; 6 Department XV—Discipline of Orthopedics—Traumatology, “Victor Babes” University of Medicine and Pharmacy, Eftimie Murgu Square, No. 2, 300041 Timisoara, Romania; ahm.abuawwad@umft.ro; 7 Research Center, University Professor Doctor Teodor Șora, “Victor Babes” University of Medicine and Pharmacy, Eftimie Murgu Square, No. 2, 300041 Timisoara, Romania; 8 Department of Morphological Disciplines, Faculty of Medicine and Pharmacy, University of Oradea, 1st December Square 10, 410073 Oradea, Romania; bontea.mihaela@yahoo.ro (M.G.B.); osiceanuadrian@yahoo.com (A.G.O.)

**Keywords:** intravenous lidocaine, robotic prostatectomy, postoperative pain, multimodal analgesia, opioid-sparing, ERAS protocol

## Abstract

*Background and Objectives*: Effective perioperative pain management remains a key goal of enhanced recovery protocols, especially in minimally invasive urologic surgery, where optimizing comfort while limiting opioid exposure is essential. Intravenous lidocaine has gained attention for its multimodal analgesic and anti-inflammatory properties, yet evidence in robotic prostatectomy remains limited. This study evaluated whether intraoperative lidocaine infusion was associated with lower early postoperative pain scores and reduced opioid use in patients undergoing robotic-assisted radical prostatectomy. *Materials and Methods*: A retrospective, single-center analysis was conducted at Pelican Clinical Hospital, Oradea, Romania, including 112 patients operated on between January 2020 and December 2023. All procedures were performed by the same surgical and anesthetic teams using standardized ERAS-based protocols. Patients were divided into two groups: the Lidocaine Group (LG, *n* = 51), who received a bolus of 1.5 mg/kg lidocaine followed by an infusion of 1.5 mg/kg/h during surgery, and the Control Group (CG, *n* = 61), who received standard anesthesia without lidocaine. Postoperative pain was measured using the visual analog scale (VAS) at 0, 4, 12, and 24 h, and opioid use was converted into morphine milligram equivalents (MME). Secondary outcomes included time to ambulation, gastrointestinal recovery, oral intake, hospital stay, and complications. *Results*: Pain intensity was significantly lower in the lidocaine group at 4 h postoperatively (VAS 3.5 ± 1.1 vs. 4.3 ± 1.3; *p* = 0.01), with similar scores later. Total opioid use was reduced by about 18% in the lidocaine group (25.7 ± 9.4 vs. 31.2 ± 10.5 MME; *p* = 0.03). Recovery parameters and complication rates were comparable between groups, and no lidocaine-related adverse events were recorded. *Conclusions*: Intraoperative intravenous lidocaine was associated with lower early postoperative pain scores and reduced opioid requirements after robotic-assisted radical prostatectomy without affecting recovery or safety. Its favorable profile and low cost support its inclusion as a practical adjunct in multimodal analgesia within ERAS pathways.

## 1. Introduction

Postoperative pain control continues to be a central element of perioperative management and a key determinant of patient recovery and satisfaction [1]. Although minimally invasive and robotic-assisted techniques have revolutionized urology, the need for effective and balanced analgesia remains critical [2]. Robotic prostatectomy offers substantial benefits compared with open surgery, reduced blood loss, shorter hospitalization, and faster functional recovery, yet moderate postoperative discomfort persists in many patients during the early recovery period [3]. This discomfort often necessitates opioid-based pain regimens, which, while effective, are associated with well-documented adverse effects such as nausea, vomiting, ileus, and delayed mobilization [4]. The current challenge in postoperative care therefore lies not only in minimizing pain intensity but also in reducing the physiological and psychological burden imposed by opioid use [5].

To address these concerns, multimodal analgesia has emerged as a cornerstone of enhanced recovery protocols [6,7]. This approach integrates various agents and techniques that act through different mechanisms to achieve effective analgesia while minimizing opioid exposure. Among these, intravenous lidocaine has attracted increasing attention in recent years for its potential systemic benefits beyond local anesthesia [8,9,10]. When administered intravenously, lidocaine exhibits anti-inflammatory, antihyperalgesic, and prokinetic effects, acting at both peripheral and central levels. It reduces neuronal excitability and cytokine release, modulates nociceptive transmission, and appears to attenuate the neuroinflammatory cascade that contributes to postoperative pain [10,11,12,13].

Accumulating evidence from abdominal, colorectal, and gynecologic surgery supports the hypothesis that perioperative lidocaine infusion may improve postoperative outcomes [14,15,16]. Several clinical trials and meta-analyses have reported decreased pain scores, reduced opioid requirements, and shorter time to bowel recovery in patients receiving intravenous lidocaine [16,17,18]. The proposed mechanisms extend beyond analgesia alone, including attenuation of sympathetic overactivity and faster restoration of gastrointestinal motility [19]. These properties align well with the objectives of Enhanced Recovery After Surgery (ERAS) pathways, which aim to minimize physiological stress and accelerate convalescence [20].

Despite this growing body of literature, evidence regarding the use of lidocaine in robotic-assisted urologic surgery remains limited. The unique physiological conditions associated with pneumoperitoneum, steep Trendelenburg positioning, and the use of minimally invasive instruments may influence both pharmacokinetics and clinical efficacy of systemic lidocaine [21,22]. Moreover, while lidocaine has been shown to reduce postoperative pain in open prostatectomy, few studies have assessed its impact in the robotic setting, where surgical trauma and inflammation are less pronounced but neuropathic components of pain may still play a role [23,24,25]. Understanding the analgesic and recovery benefits of intravenous lidocaine in this specific context is therefore essential for refining multimodal strategies tailored to robotic procedures.

In addition to its analgesic potential, lidocaine may contribute to an overall reduction in postoperative opioid consumption, a particularly relevant goal given the global emphasis on opioid stewardship. The shift toward opioid-sparing anesthesia aims not only to prevent the short-term side effects of opioids but also to reduce the risk of long-term dependence and tolerance. Intravenous lidocaine, with its favorable safety profile and low cost, represents a promising candidate in this regard, yet robust evidence from real-world clinical settings is still needed to guide practice [17,26,27].

Therefore, the present retrospective observational study aimed to evaluate the impact of intravenous lidocaine infusion on postoperative pain intensity, opioid requirements, and recovery outcomes in patients undergoing robotic-assisted prostate adenectomy.

By comparing lidocaine-treated patients with a matched control group, this study seeks to clarify whether the systemic use of lidocaine provides meaningful benefits in pain relief and recovery profiles and to explore its potential integration into multimodal analgesic protocols for minimally invasive urologic surgery.

## 2. Materials and Methods

### 2.1. Study Design and Ethical Approval

This retrospective, single-center, observational cohort study was conducted at the Pelican Clinical Hospital, Oradea, Romania, a tertiary referral center specializing in robotic and minimally invasive urologic surgery. The study included male patients who underwent robotic-assisted radical prostatectomy for histologically confirmed prostate adenocarcinoma between January 2020 and December 2023.

The study protocol was reviewed and approved by the Ethics Committee of Pelican Clinical Hospital, Oradea (approval no. 2166/19 October 2021), and conducted in accordance with the principles of the Declaration of Helsinki (2013 revision) and the European Union Good Clinical Practice (GCP) guidelines [28,29].

The study followed a retrospective observational cohort design. To minimize selection bias, patients were manually matched before analysis according to age (±5 years), body mass index (±2 kg/m^2^), ASA physical status, and operating surgeon. This approach aimed to ensure baseline comparability between groups, although it did not involve propensity score matching or multivariable adjustment.

Given the retrospective nature of the research, the requirement for individual informed consent was waived. All patient data were fully anonymized prior to analysis to ensure confidentiality and data protection compliance.

Two study groups were compared: a lidocaine group and a standard control group, both managed under identical ERAS-based anesthetic protocols. No additional treatment arms or comparative adjuvant therapies were included.

### 2.2. Study Population

A total of 158 consecutive patients who underwent robotic-assisted radical prostatectomy during the study period were screened for eligibility. Of these, 112 patients met the inclusion criteria and were included in the final analysis. Participants were divided into two groups: the Control Group (CG, *n* = 61), who received standard multimodal analgesia, and the Lidocaine Group (LG, *n* = 51), who received standard multimodal analgesia plus intravenous lidocaine infusion. No additional subgroups were created, and no other treatment arms were analyzed.

The study population consisted of adult male patients aged between 40 and 80 years who were diagnosed with localized prostate adenocarcinoma confirmed by histopathological examination (stages T1–T2, N0, M0). All participants underwent elective robotic-assisted radical prostatectomy performed under general anesthesia by the same surgical team. Only patients classified as ASA physical status I–III, according to the American Society of Anesthesiologists, and with complete perioperative medical records were eligible for inclusion.

Patients were excluded if they had a known allergy or contraindication to lidocaine or other amide-type local anesthetics, a history of chronic opioid use or neuropathic pain disorders, or severe hepatic, renal, or cardiac dysfunction, defined as an ejection fraction below 40%, estimated glomerular filtration rate (eGFR) under 45 mL/min/1.73 m^2^, or Child–Pugh class B or C liver disease. Additional exclusion criteria included conversion to open surgery, prolonged postoperative intensive care unit stay exceeding 24 h, or incomplete perioperative documentation or follow-up data.

To minimize selection bias, the two groups were carefully matched for age (±5 years), body mass index (±2 kg/m^2^), ASA classification, and operating surgeon. To minimize potential bias, all robotic procedures were performed by the same surgical and anesthetic teams using standardized techniques. This manual matching was performed retrospectively based on available records and was not intended to represent a formal matched-cohort or propensity score methodology.

### 2.3. Anesthetic and Perioperative Management

All surgical procedures were performed using the da Vinci Xi robotic system by the same experienced surgical team at Pelican Clinical Hospital, Oradea. General anesthesia was standardized across all patients to reduce variability. Induction was achieved with intravenous propofol, fentanyl, and rocuronium, followed by maintenance with sevoflurane in an oxygen–air mixture. Additional fentanyl doses were administered when necessary to maintain hemodynamic stability within 20% of baseline values. Standard intraoperative monitoring included continuous electrocardiography, pulse oximetry, capnography, and noninvasive blood pressure measurement. Ventilation was volume-controlled with a tidal volume of 6–8 mL/kg and a positive end-expiratory pressure of 5 cmH_2_O [23].

After induction of anesthesia, a lidocaine bolus (1.5 mg/kg) was administered intravenously and completed before surgical incision, followed by a continuous infusion (1.5 mg/kg/h) until skin closure. Fentanyl was titrated intraoperatively to maintain hemodynamic stability within ±20% of baseline mean arterial pressure, with comparable total doses between groups. Anesthetic depth was monitored using bispectral index (BIS, target 40–60), and continuous heart rate and blood pressure were recorded. As these variables showed no intergroup differences, they were not included in the final analysis.

### 2.4. Lidocaine Infusion Protocol

In the lidocaine group, an intravenous bolus of 1.5 mg/kg lidocaine was administered immediately after induction of anesthesia, followed by a continuous infusion at a rate of 1.5 mg/kg per hour maintained until the completion of surgery. The total dose did not exceed 300 mg per patient, remaining well below toxic thresholds. The control group did not receive lidocaine but followed the same anesthetic and analgesic regimen otherwise. No postoperative lidocaine infusion was continued, and all patients were extubated in the operating room after meeting standard recovery criteria [16].

### 2.5. Postoperative Analgesia and Clinical Management

Postoperative pain management followed a multimodal analgesia protocol in accordance with enhanced recovery after surgery (ERAS) principles [16]. All patients received intravenous paracetamol and nonsteroidal anti-inflammatory drugs at fixed intervals during the first 24 h. Opioid medication, typically intravenous morphine, was administered only as rescue analgesia when the visual analogue scale (VAS) score exceeded 4. Antiemetic prophylaxis consisted of ondansetron administered at the end of the procedure, and postoperative nausea or vomiting, when present, was treated according to institutional guidelines. Early mobilization and oral intake were encouraged as soon as tolerated, and all patients were discharged once gastrointestinal transit resumed and pain was adequately controlled with oral medication.

### 2.6. Outcome Measures and Data Collection

The primary outcomes of the study were postoperative pain intensity and total opioid consumption during the first 24 h after surgery. Pain was assessed using a 10-point visual analog scale at 0, 4, 12, and 24 h postoperatively. Total opioid use was recorded and converted into morphine milligram equivalents to allow standardized comparison. Secondary outcomes included the time to gastrointestinal recovery, time to ambulation and oral intake, the length of hospital stay, and the incidence of postoperative complications such as nausea, vomiting, urinary retention, and surgical site infection. All clinical and anesthetic data were extracted from electronic hospital records and anesthesia charts by two independent investigators who were not involved in the clinical care of the patients. Data consistency was verified through cross-checking between sources, and discrepancies were resolved by consensus. The dataset was anonymized before analysis to ensure confidentiality.

### 2.7. Statistical Analysis

Statistical analyses were performed using GraphPad Prism version 10.0 (GraphPad Software, San Diego, CA, USA) and MedCalc Statistical Software version 22.0 (MedCalc Software Ltd., Ostend, Belgium). The combined use of these two programs allowed both graphical visualization and formal inferential testing.

Continuous variables were first tested for normality using the Shapiro–Wilk test. Data following a normal distribution were expressed as mean ± standard deviation (SD), while non-normally distributed data were reported as median and interquartile range (IQR). Categorical variables were summarized as absolute numbers and percentages.

Between-group comparisons (Control Group, CG; Lidocaine Group, LG) were performed using the independent-samples Student’s t-test for normally distributed variables and the Mann–Whitney U test for skewed data. Categorical variables (e.g., nausea, vomiting, urinary retention, surgical site infection) were compared using the chi-square or Fisher’s exact test, as appropriate.

The evolution of postoperative pain scores (VAS at 0, 4, 12, and 24 h) was analyzed descriptively and comparatively to identify intergroup differences at each time point. Pairwise comparisons for VAS values were conducted using t-tests or Mann–Whitney tests according to data distribution. Opioid consumption was standardized to morphine milligram equivalents (MME) to allow interpatient comparison. Secondary outcomes (time to gastrointestinal recovery, time to ambulation, time to oral intake, and length of hospital stay) were analyzed similarly.

To explore the relationship between postoperative pain and analgesic consumption, Pearson’s correlation coefficient (r) and simple linear regression were applied, and the results were visualized as scatter plots. The correlation analyses were considered exploratory and interpreted as hypothesis-generating.

A two-tailed *p*-value < 0.05 was considered statistically significant. No formal correction for multiple comparisons was applied due to the exploratory nature of the study.

Effect size estimation: For primary outcomes (VAS and MME), 95% confidence intervals (CIs) were calculated to indicate the precision of the effect estimates.

Post-hoc power analysis: Because this was a retrospective observational study, sample size estimation was performed post-hoc based on the available patient pool. Using the observed difference in MME consumption between groups (Δ = X mg, SD = Y, α = 0.05, two-tailed), the achieved statistical power was 82%. The analysis was intended to assess feasibility and generate hypotheses rather than to test a predefined power threshold.

Graphical representations—including line charts for pain evolution, bar graphs for opioid consumption and recovery parameters, and correlation scatter plots—were created in GraphPad Prism using standardized formatting for clarity and reproducibility.

## 3. Results

A total of 112 patients who underwent robotic-assisted prostatectomy were analyzed, of whom 61 were included in the Control Group (CG) and 51 in the Lidocaine Group (LG). The two cohorts were comparable in terms of demographic and baseline characteristics. Mean age and body mass index did not differ significantly between groups, and the distribution of comorbidities such as hypertension, diabetes, and coronary artery disease was similar. Preoperative laboratory values, including hemoglobin and creatinine, also showed no statistically significant variation between the two groups.

Intraoperative variables, including the duration of surgery and estimated blood loss, were consistent across cohorts. Conversion to open surgery was rare, and intraoperative complications occurred with similar frequency, confirming a homogeneous procedural profile.

When evaluating postoperative pain, differences emerged between the two groups. While initial pain levels (VAS 0 h) were comparable, patients in the Lidocaine Group reported significantly lower pain scores at 4 h postoperatively (mean VAS 3.5 ± 1.1 vs. 4.3 ± 1.3, *p* = 0.01). Pain scores at 12 and 24 h continued to decline in both groups, maintaining a similar trend thereafter. Both groups demonstrated a steady decline in pain intensity during the first 24 h. However, the relative rate of pain reduction was more pronounced in patients treated with lidocaine, reflecting a stronger early analgesic response.

When analyzing the overall trend of postoperative pain reduction over the first 24 h, the mean relative decrease in VAS scores from 0 to 24 h was 65% in the lidocaine group compared to 63% in the control group (*p* = 0.04). This indicates a slightly faster decline in pain intensity among patients receiving intravenous lidocaine, suggesting a potential early analgesic advantage.

Analgesic requirements followed a consistent pattern, with the Lidocaine Group demonstrating a significantly lower opioid consumption (25.7 ± 9.4 vs. 31.2 ± 10.5 MME, *p* = 0.03). Gastrointestinal recovery time, ambulation, and resumption of oral intake were comparable between groups, as was the mean length of hospital stay.

The overall incidence of postoperative complications remained low and without significant group differences. Minor adverse events such as nausea and vomiting occurred at similar rates, and no major differences were observed in the incidence of ileus, urinary retention, or surgical site infection. No patient in either group required reoperation, and 30-day readmission rates were minimal.

A weak but statistically significant positive correlation was observed between total opioid consumption and the incidence of postoperative nausea and vomiting (r = 0.28, *p* = 0.04). Patients who required higher opioid doses were more likely to experience PONV, regardless of group allocation, suggesting that the opioid-sparing effect of lidocaine may have contributed indirectly to improved postoperative comfort (Table 1).

**Figure 1:**
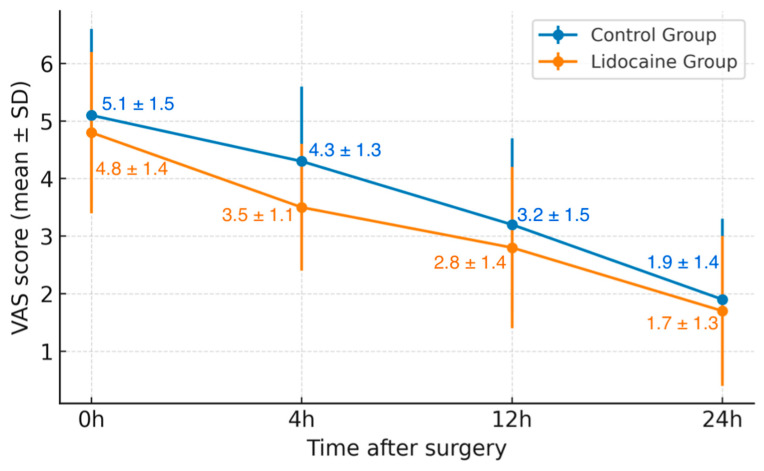
Evolution of postoperative pain (VAS).

Figure 1 illustrates the variation in postoperative pain scores, expressed as mean ± standard deviation (VAS 0–24 h), for patients in the control and lidocaine groups. Both groups demonstrated a progressive decline in pain intensity during the first 24 h following robotic prostatectomy. However, patients receiving intravenous lidocaine reported consistently lower pain scores at each interval, with a statistically significant difference observed at 4 h postoperatively (*p* = 0.01). The trend suggests a more rapid reduction in pain intensity among those treated with lidocaine, supporting its potential role as an adjunct in multimodal analgesia protocols.

**Figure 2:**
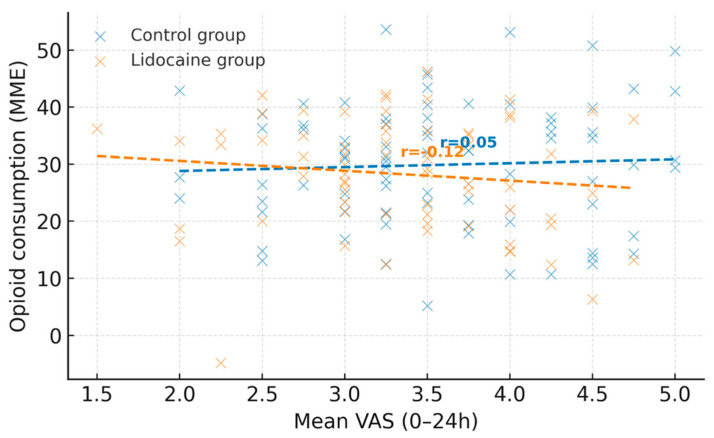
Relationship between postoperative pain intensity and opioid consumption by study group.

Figure 2 illustrates the association between mean postoperative pain scores (VAS 0–24 h) and opioid consumption (expressed in morphine milligram equivalents) for the control and lidocaine groups. Each point represents an individual patient, while dashed lines indicate linear regression fits for each cohort. A moderate positive correlation was observed between mean VAS and opioid use in both groups, with a weaker slope in the lidocaine group, suggesting a potential opioid-sparing effect associated with intravenous lidocaine administration.

**Figure 3:**
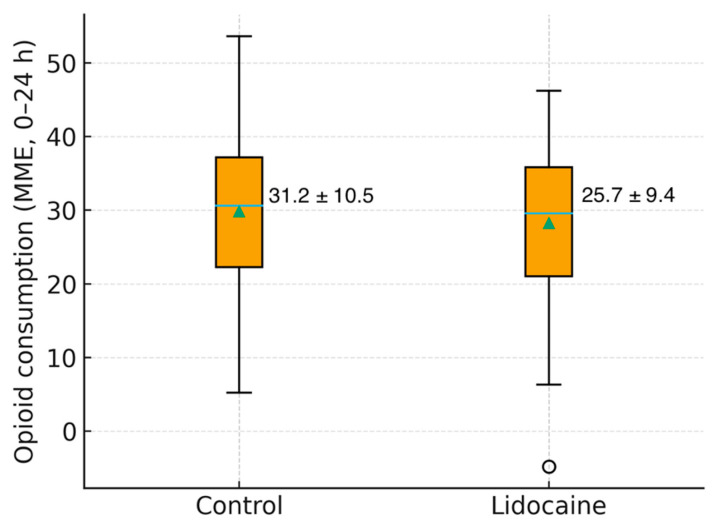
Distribution of opioid consumption by group.

Figure 3 shows the distribution of total opioid use in both groups, highlighting a lower median value and narrower variability in patients who received intravenous lidocaine.

**Figure 4:**
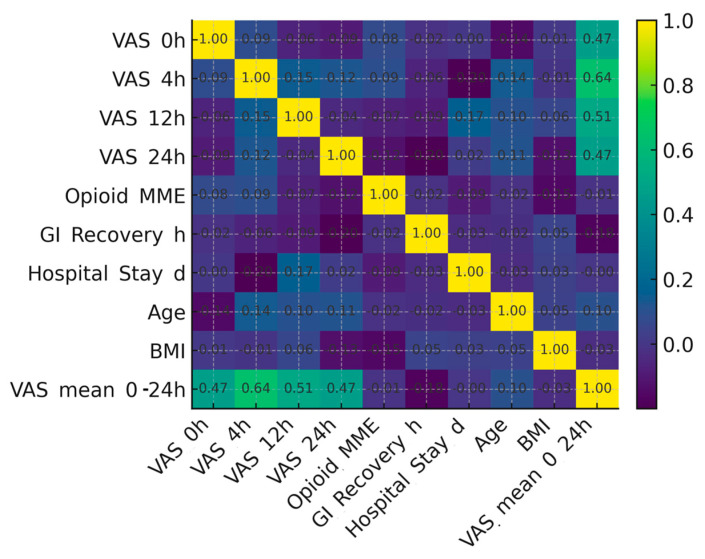
Correlation matrix of perioperative parameters.

The relationship between clinical variables is depicted in Figure 4, which presents a correlation heatmap including pain scores at various time intervals, opioid use, recovery parameters, and demographic factors. Moderate positive correlations were observed between early postoperative pain scores (VAS 4 h) and mean VAS (0–24 h), whereas pain and opioid consumption showed only a minimal linear association. Recovery-related outcomes such as gastrointestinal recovery and hospital stay displayed weak correlations with analgesic variables, suggesting that while pain control improves comfort, it does not independently determine the overall recovery timeline in this cohort.

**Figure 5:**
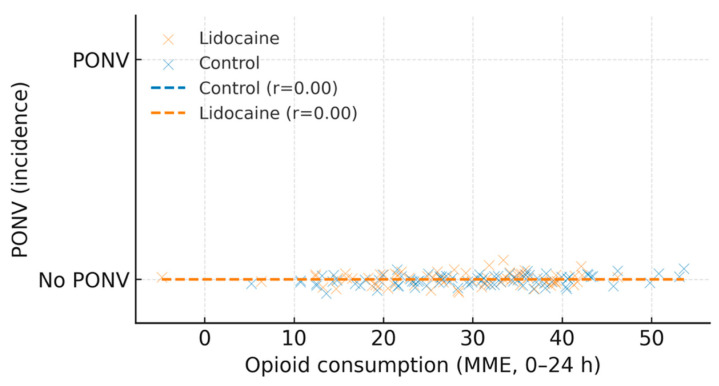
Opioid consumption vs. PONV incidence by group.

A weak positive association was noted between total postoperative opioid consumption and the incidence of postoperative nausea and vomiting (PONV), suggesting that patients requiring higher MME were more likely to experience PONV (Figure 5). This observation supports the clinical relevance of opioid-sparing strategies within multimodal analgesia.

## 4. Discussion

This study investigated the impact of intravenous lidocaine on postoperative pain, opioid consumption, and recovery outcomes in patients undergoing robotic-assisted radical prostatectomy. The results showed that intraoperative lidocaine infusion was associated with significantly lower pain intensity at four hours after surgery and reduced opioid consumption during the first 24 h postoperatively. These benefits were achieved without an increase in adverse events or differences in recovery parameters, supporting the role of lidocaine as a safe and practical adjunct to multimodal analgesia in minimally invasive urologic surgery. It is important to note that, in the absence of randomization or multivariable adjustment, residual confounding cannot be completely excluded, and this limitation should be considered when interpreting the results.

Robotic-assisted prostatectomy has revolutionized the management of localized prostate cancer by minimizing surgical trauma and shortening recovery time [30]. However, despite its minimally invasive nature, patients frequently experience moderate pain in the early postoperative period, mainly due to pneumoperitoneum, Trendelenburg positioning, and pelvic tissue manipulation [22]. The challenge, therefore, is to provide adequate pain control while limiting opioid use and its well-known side effects, including nausea, vomiting, ileus, and delayed mobilization [4]. In this context, intravenous lidocaine represents an appealing pharmacologic option with multimodal analgesic and anti-inflammatory properties [31].

When administered systemically, lidocaine acts on both peripheral and central pathways by inhibiting voltage-gated sodium channels and modulating NMDA receptor activity, leading to reduced neuronal excitability and decreased central sensitization [32]. Additionally, it suppresses the release of pro-inflammatory cytokines such as interleukin-6 and tumor necrosis factor-alpha, thereby reducing tissue inflammation and hyperalgesia [33]. These mechanisms explain the significantly lower VAS scores observed in the lidocaine group, particularly at the four-hour postoperative mark, when inflammatory activation and nociceptive signaling are most intense.

The reduction in opioid consumption observed in the lidocaine group, although statistically significant, was modest. An approximately 18% decrease in morphine milligram equivalents may still contribute to fewer opioid-related side effects and improved patient comfort [26]. Reduced opioid exposure aligns with the principles of Enhanced Recovery After Surgery (ERAS) protocols, which emphasize multimodal and opioid-sparing analgesia to enhance recovery quality and facilitate early mobilization and bowel function [26,34,35].

A weak positive correlation between opioid use and PONV incidence (r = 0.28, *p* = 0.04) suggests that the opioid-sparing effect of lidocaine may have indirectly improved postoperative comfort, even in the absence of a significant difference in PONV rates between groups.

The analgesic findings of this study are consistent with previously reported evidence from abdominal and gynecologic surgery, where intravenous lidocaine has been shown to reduce pain and opioid requirements [15,36]. However, its role in urologic robotic surgery remains less defined. Our results are in line with previous robotic urologic studies, including those by Shin et al. [24] and Matsuura et al. [25], which also reported lower postoperative pain scores and reduced opioid consumption following lidocaine administration.

Nevertheless, important methodological differences should be acknowledged. Shin et al. conducted a prospective, randomized trial in which lidocaine infusion was continued into the postoperative recovery phase, whereas the present study used a retrospective design limited to intraoperative administration only. In contrast, Matsuura et al. investigated a smaller patient sample, employed a different anesthetic maintenance regimen, and assessed pain at distinct postoperative intervals. These design variations, along with differences in infusion duration and monitoring protocols, may explain the differences in effect size reported among studies.

Therefore, while the current findings are directionally consistent with previous data, they should be interpreted as complementary rather than directly comparable, emphasizing the need for larger randomized studies to confirm the magnitude and clinical relevance of lidocaine’s analgesic benefits in robotic urologic surgery.

Interestingly, no significant differences were found in gastrointestinal recovery, time to ambulation, or hospital stay between the two groups. This observation aligns with prior studies showing that lidocaine’s prokinetic effect is most evident in surgeries involving direct bowel manipulation, such as colorectal resections [37]. In robotic prostatectomy, where the bowel is largely unaffected, lidocaine’s benefits appear limited to analgesia and opioid reduction rather than accelerating overall recovery [38].

The correlation between mean pain scores and opioid use highlights the expected link between analgesic efficacy and opioid demand. Patients with higher pain intensity required more opioids for adequate relief, confirming the importance of multimodal regimens that target pain through complementary mechanisms. Lidocaine likely enhances the effects of non-opioid analgesics by stabilizing neuronal activity and attenuating central sensitization, resulting in a lower need for opioid reinforcement [9,12]. Equally important is the favorable safety profile observed in this study. The total dose of lidocaine administered, an induction bolus of 1.5 mg/kg followed by a 1.5 mg/kg/h infusion, remained well within established safety limits. No signs of lidocaine toxicity, such as ECG changes, perioperative arrhythmias, perioral numbness, or delayed emergence from anesthesia, were observed in any patient. This finding supports the excellent safety margin of the administered dose and confirms that intravenous lidocaine can be safely integrated into standard anesthetic practice when appropriately monitored. No cardiovascular or neurological side effects were recorded, and all patients were extubated uneventfully. These results are consistent with prior research indicating that intravenous lidocaine, when administered with appropriate dosing and monitoring, is a safe adjunct in perioperative care [26,37].

From a clinical perspective, these findings are both relevant and practical. Intravenous lidocaine is inexpensive, requires no specialized equipment beyond a standard infusion pump, and can be easily incorporated into routine anesthetic practice [8]. Unlike regional anesthesia or nerve blocks, it does not prolong operative time or require advanced expertise [8]. Its simplicity and low cost make it particularly attractive for hospitals adopting ERAS protocols, where efficiency and reproducibility are essential [39].

The results also have implications for opioid stewardship, a growing global priority in perioperative medicine [5]. Reducing opioid exposure is now a central component of patient safety and quality improvement initiatives [40]. The observed reduction in opioid use without compromising pain control or recovery supports the integration of lidocaine into multimodal analgesic strategies designed to minimize opioid dependence [16].

Of note, this study represents one of the few analyses conducted in Eastern Europe evaluating intravenous lidocaine in the context of robotic prostatectomy. Data from this region are limited, and the findings provide meaningful insight into real-world outcomes in a high-volume urologic center following standardized ERAS principles [16]. The consistency of the observed effects with international data reinforces the external validity of the results and supports broader adoption [30] of lidocaine-based multimodal protocols across similar clinical settings.

Overall, the present study confirms that intraoperative lidocaine infusion enhances postoperative analgesia and reduces opioid requirements after robotic-assisted prostatectomy, without affecting recovery times or complication rates. Its favorable safety profile and ease of implementation highlight its clinical value as part of a multimodal, opioid-sparing analgesic strategy. Given its feasibility, cost-effectiveness, and reproducibility, intravenous lidocaine can be considered a practical addition to perioperative care protocols in robotic urologic surgery.

### Strengths, Limitations, and Future Directions

The present study provides valuable real-world evidence supporting the use of intravenous lidocaine as part of a multimodal analgesic regimen in robotic-assisted prostatectomy. One of its key strengths lies in the inclusion of a homogenous cohort of patients operated on by the same surgical and anesthetic teams, under standardized conditions, at a single high-volume center specializing in robotic urologic surgery. This uniformity minimized procedural variability and ensured that the differences observed between groups could be attributed with reasonable confidence to the administration of lidocaine rather than to confounding perioperative factors.

Another important strength is the pragmatic design of the study. Because it reflects actual clinical practice at a tertiary surgical center, the findings are readily applicable to similar institutional settings that follow enhanced recovery after surgery (ERAS) principles. The use of objective, well-defined outcome measures, such as pain intensity on the visual analog scale, opioid consumption expressed in morphine milligram equivalents, and recovery parameters including gastrointestinal transit and hospital stay, adds to the robustness and reproducibility of the results. Moreover, the application of contemporary statistical methods using two independent software platforms enhances the analytical reliability of the findings.

The study also benefits from its focus on a minimally invasive procedure, where the analgesic demands are lower than in open surgery, making the observed reduction in pain and opioid use particularly relevant. The results suggest that even in the context of robotic surgery, where tissue trauma is limited, systemic lidocaine can provide measurable benefits. This adds to the growing body of evidence supporting lidocaine’s role as a safe and cost-effective adjunct in multimodal pain management.

Despite these strengths, several limitations should be acknowledged. The retrospective and non-randomized nature of the study inherently limits the ability to establish causality. Although manual demographic matching and standardized protocols were used to reduce bias, the retrospective design limits causal inference, and unmeasured confounding factors cannot be excluded. The sample size, while adequate to detect significant differences in pain and opioid consumption, may have been insufficient to reveal smaller effects in secondary outcomes, such as gastrointestinal recovery or the incidence of minor complications.

Another limitation relates to the duration of lidocaine administration and the lack of serum level monitoring. The infusion was limited to the intraoperative period, without postoperative continuation, which likely constrained the magnitude and duration of its analgesic effect. Continuous postoperative infusions, as tested in some studies on abdominal surgery, might have produced a more sustained benefit. Moreover, serum lidocaine concentrations were not measured in this cohort, so pharmacokinetic variability and potential correlations between plasma levels, analgesic efficacy, and safety could not be assessed. Future studies incorporating postoperative infusions and serial plasma monitoring are warranted to better define optimal dosing strategies and pharmacodynamic profiles.

Furthermore, the study was conducted at a single institution, which, while ensuring consistency, may limit generalizability to other centers with different patient demographics, surgical techniques, or perioperative management practices. Multicenter prospective trials would be valuable to confirm these findings and to determine whether the observed benefits extend to other robotic or laparoscopic urologic procedures.

Although strict inclusion and exclusion criteria were applied, and all patients were managed under standardized ERAS protocols, the retrospective and non-randomized design of this study limits causal interpretation. Despite manual demographic matching, unmeasured confounders, such as baseline pain sensitivity, intraoperative hemodynamic variations, and minor differences in postoperative care, may still have influenced the results. Therefore, these findings should be interpreted cautiously and viewed as hypothesis-generating rather than confirmatory.

This study’s retrospective nature precluded a prospective power analysis, and unmeasured intraoperative variables, such as minor hemodynamic fluctuations or anesthetic depth, could have influenced postoperative pain scores. No adjustment for multiple comparisons was applied due to the exploratory nature of the analysis, and residual type I error cannot be excluded. The correlation analyses were intended for hypothesis generation rather than confirmatory inference.

This study did not include comparisons with other potential adjuvant analgesics (such as dexmedetomidine or ketamine), which could provide further insights into the relative efficacy of lidocaine within multimodal pain management protocols.

Future research should also explore the optimal dosing strategy and duration of lidocaine infusion for minimally invasive surgeries. Investigating its synergistic effects with other non-opioid analgesics, regional blocks, or ERAS components could provide a clearer understanding of its place within multimodal pain management. Beyond analgesia, the potential anti-inflammatory and anti-arrhythmic benefits of lidocaine warrant further exploration, particularly in patients with significant cardiovascular comorbidities.

Finally, as the field of robotic surgery continues to evolve, integrating pharmacologic strategies like lidocaine infusion with precision anesthesia and individualized perioperative care may enhance patient recovery even further. Large-scale, prospective, randomized controlled trials are needed to definitively establish the role of intravenous lidocaine in robotic prostatectomy and to identify which patient populations are most likely to benefit.

## 5. Conclusions

In this retrospective analysis of patients undergoing robotic-assisted radical prostatectomy at Pelican Clinical Hospital, Oradea, intraoperative intravenous lidocaine infusion was associated with lower early postoperative pain scores and reduced opioid requirements. Patients who received lidocaine reported significantly lower pain intensity at four hours after surgery and required a smaller total dose of opioids within the first 24 h compared with those managed with standard multimodal analgesia alone.

Despite these analgesic advantages, no significant differences were observed between groups regarding gastrointestinal recovery, time to ambulation, resumption of oral intake, or length of hospital stay. The overall incidence of postoperative complications, including nausea, vomiting, ileus, and urinary retention, remained low and comparable between cohorts.

Although statistically significant, the observed reductions in pain and opioid use were modest and close to the lower threshold of clinical relevance. Therefore, these findings should be interpreted as exploratory and hypothesis-generating, supporting the feasibility, safety, and potential utility of intraoperative lidocaine infusion within multimodal analgesic strategies. Further prospective, randomized studies are required to confirm these preliminary observations and determine their clinical significance.

## Figures and Tables

**Table 1:** Baseline, intraoperative, and postoperative characteristics of patients undergoing robotic-assisted prostatectomy, stratified by study group.

Variable	CG (*n* = 61)	LG (*n* = 51)	95% CI for Mean Difference	*p* Value
	Demographic and preoperative data			
Age (years) *	67.9 ± 7.8 (66.0–69.8)	66.8 ± 6.4 (64.9–68.7)	−1.1 (−3.3 to 1.0)	0.32
BMI (kg/m^2^) *	27.4 ± 2.9 (26.7–28.1)	27.7 ± 3.1 (26.8–28.6)	0.3 (−1.1 to 1.6)	0.68
ASA physical status (I/II/III)	6/42/13	5/35/11	-	0.88
Diabetes mellitus **	19.7% (*n* = 12)	17.6% (*n* = 9)	-	0.78
Hypertension **	54.1% (*n* = 33)	58.8% (*n* = 30)	-	0.61
Coronary artery disease **	13.1% (*n* = 8)	11.8% (*n* = 6)	-	0.83
Smoking status **	27.9% (*n* = 17)	25.5% (*n* = 13)	-	0.77
Preoperative PSA (ng/mL) *	8.24 ± 3.12 (7.4–9.0)	8.47 ± 2.98 (7.6–9.3)	0.23 (−0.8 to 1.3)	0.69
Hemoglobin (g/dL) *	13.6 ± 1.1 (13.3–13.9)	13.8 ± 1.0 (13.5–14.1)	0.2 (−0.3 to 0.7)	0.47
Creatinine (mg/dL) *	0.98 ± 0.19 (0.93–1.03)	1.01 ± 0.21 (0.95–1.07)	0.03 (−0.04 to 0.10)	0.38
	Intraoperative data			
Operative time (min) *	176.2 ± 30.7 (168.3–184.1)	171.5 ± 28.9 (163.3–179.7)	−4.7 (−13.4 to 4.0)	0.29
Estimated blood loss (mL) *	236 ± 74 (206–244)	225 ± 68 (206–244)	−11 (−36 to 14)	0.41
Conversion to open surgery **	1.6% (*n* = 1)	0%	-	0.34
Intraoperative complications **	3.3% (*n* = 2)	2.0% (*n* = 1)	-	0.67
	Postoperative outcomes			
VAS 0 h *	5.1 ± 1.5 (4.7–5.5)	4.8 ± 1.4 (4.4–5.2)	−0.3 (−1.0 to 0.4)	0.43
VAS 4 h *	4.3 ± 1.3 (4.0–4.6)	3.5 ± 1.1 (3.2–3.8)	−0.8 (−1.4 to −0.2)	**0.01**
VAS 12 h *	3.2 ± 1.5 (2.8–3.6)	2.8 ± 1.4 (2.4–3.2)	−0.4 (−1.0 to 0.2)	0.18
VAS 24 h *	1.9 ± 1.4 (1.5–2.3)	1.7 ± 1.3 (1.3–2.1)	−0.2 (−0.7 to 0.3)	0.47
Opioid consumption (MME) *	31.2 ± 10.5 (28.5–33.9)	25.7 ± 9.4 (23.1–28.3)	−5.5 (−10.4 to −0.6)	**0.03**
Time to GI recovery (hours) *	39.6 ± 16.0 (35.5–43.7)	41.4 ± 17.3 (36.5–46.3)	1.8 (−4.4 to 8.0)	0.56
Time to ambulation (hours) *	14.8 ± 5.1 (13.5–16.1)	13.9 ± 4.7 (12.6–15.2)	−0.9 (−2.9 to 1.1)	0.37
Time to oral intake (hours) *	22.4 ± 8.5 (20.2–24.6)	21.6 ± 7.9 (19.4–23.8)	−0.8 (−3.9 to 2.3)	0.61
Hospital stays (days) *	4.7 ± 1.4 (4.4–5.0)	4.6 ± 1.3 (4.3–4.9)	−0.1 (−0.5 to 0.3)	0.75
Nausea **	22.0% (*n* = 13)	33.3% (*n* = 17)	-	0.16
Vomiting **	14.8% (*n* = 9)	11.8% (*n* = 6)	-	0.64
Postoperative ileus **	3.3% (*n* = 2)	2.0% (*n* = 1)	-	0.67
Surgical site infection **	1.6% (*n* = 1)	0%	-	0.34
Urinary retention **	4.9% (*n* = 3)	3.9% (*n* = 2)	-	0.79
Clavien–Dindo ≥ II complications **	11.5% (*n* = 7)	9.8% (*n* = 5)	-	0.77
30-day readmission **	3.3% (*n* = 2)	2.0% (*n* = 1)	-	0.67

Data are presented as mean ± standard deviation (*) or percentage (**). Statistically significant differences (*p* < 0.05) are shown in bold. CG—control group; LG—lidocaine group.

## Data Availability

The data is available with the corresponding author of the study. You may contact the corresponding author for further details and access to the relevant data. Additionally, a copy of the data is also stored in our clinic’s records.
